# Tree Shrew Is a Suitable Animal Model for the Study of Epstein Barr Virus

**DOI:** 10.3389/fimmu.2021.789604

**Published:** 2022-01-17

**Authors:** Wei Xia, Honglin Chen, Yiwei Feng, Nan Shi, Zongjian Huang, Qingyuan Feng, Xu Jiang, Guangyao He, Mao Xie, Yongjin Lai, Zhi Wang, Xiang Yi, Anzhou Tang

**Affiliations:** ^1^ Department of Otorhinolaryngology Head and Neck Surgery, The First Affiliated Hospital of Guangxi Medical University, Nanning, China; ^2^ Key Laboratory of Early Prevention and Treatment for Regional High Frequency Tumor (Gaungxi Medical University), Ministry of Education, Nanning, China

**Keywords:** Epstein-Barr virus, tree shrew, RNA-seq, neutrophils, primary infection

## Abstract

Epstein-Barr virus (EBV) is a human herpesvirus that latently infects approximately 95% of adults and is associated with a spectrum of human diseases including Infectious Mononucleosis and a variety of malignancies. However, understanding the pathogenesis, vaccines and antiviral drugs for EBV-associated disease has been hampered by the lack of suitable animal models. Tree shrew is a novel laboratory animal with a close phylogenetic relationship to primates, which is a critical advantage for many animal models for human disease, especially viral infections. Herein, we first identified the key residues in the CR2 receptor that bind the gp350 protein and facilitate viral entry. We found that tree shrew shares 100% sequence identity with humans in these residues, which is much higher than rabbits (50%) and rats (25%). *In vitro* analysis showed that B lymphocytes of tree shrews are susceptible to EBV infection and replication, as well as EBV-enhanced cell proliferation. Moreover, results of *in vivo* experiments show that EBV infection in tree shrews resembles EBV infection in humans. The infected animals exhibited transient fever and loss of weight accompanied by neutropenia and high viremia levels during the acute phase of the viral infection. Thereafter, tree shrews acted as asymptomatic carriers of the virus in most cases that EBV-related protein could be detected in blood and tissues. However, a resurgence of EBV infection occurred at 49 dpi. Nanopore transcriptomic sequencing of peripheral blood in EBV-infected animals revealed the dynamic changes in biological processes occurring during EBV primary infection. Importantly, we find that neutrophil function was impaired in tree shrew model as well as human Infectious Mononucleosis datasets (GSE85599 and GSE45918). In addition, retrospective case reviews suggested that neutropenia may play an important role in EBV escaping host innate immune response, leading to long-term latent infection. Our findings demonstrated that tree shrew is a suitable animal model to evaluate the mechanisms of EBV infection, and for developing vaccines and therapeutic drugs against EBV.

## Introduction

Epstein-Barr virus (EBV) is a ubiquitous gamma herpesvirus that latently resides in more than 90% of the world population and is associated with a variety of human diseases (1). Acute, primary EBV infection is the most common cause of Infectious Mononucleosis (IM), a febrile syndrome (2). Eventually, the host immune response is able to control the acute viremia, but is unable to completely clear latent infection in a small number of peripheral blood B cells where EBV persists for life (3). In a vast majority of individuals, latent EBV infection is asymptomatic, but in patients with congenital or acquired immunodeficiencies, the loss of immune control can result in EBV-driven proliferation of B cells into malignant lymphomas (4). EBV infection is also associated with the development of epithelial and B cell malignancies in immunocompetent hosts where every tumor cell is EBV infected, e.g., nasopharyngeal carcinoma, gastric carcinoma, Burkitt Lymphoma, and Hodgkin Lymphoma (5). It is estimated that there are over 200,000 novel EBV-related cancer cases and approximately 140,000 deaths attributed to EBV-associated malignancies, annually (6). As a result, EBV has been categorized as a Class I carcinogen by the World Health Organization (WHO) (1).

Despite the public health significance of EBV-related disease, there are still no effective vaccines and antiviral drugs. A major obstacle is the lack of validated animal models that recapitulate human infection due to its strict host range (7). The EBV gp350/220 membrane glycoprotein binds to the EBV receptor on B cells, CR2 (also known as CD21), which is essential for the process of infection and contributes to the cellular host range of EBV (8). Nonhuman primates (NHPs) seem to be a reasonable option for EBV infection animal models given that they are evolutionary related. However, the majority of NHPs are naturally infected with a kind of lymphocryptovirus (LCV) that are immunologically cross-reactive with EBV. Therefore, the natural susceptibility of NHPs to EBV infection is poor (9). Furthermore, rodents B cells do not tolerate EBV infection and replication making it difficult to establish small animal models for EBV infection. Humanized mice can be experimentally infected with EBV, but the human immune system developed in humanized mice is not fully functional, particularly the poor development of mucosa associated lymphoid tissues (10). This makes it difficult to explore complex networks of interactions between multiple immune components and EBV infection.

The chinese tree shrew (Tupaia belangeri chinensis) is a small mammalian species similar in appearance to squirrels, but more genetically closer to primates than rodents (11). Numerous studies have demonstrated that the tree shrew is a practical small animal model for studies on a variety of human viruses (12). Notably, HSV-1 and HSV-2 belonging to the same Herpesviridae as EBV, can latently infect the sensory neurons of the peripheral nervous system, and the reactivation of these viruses leads to recurring cold sores in tree shrews (13, 14). In addition, the tree shrew is the only small animal having an intact immune system that can be infected by HCV without genetic modification (15, 16). To avoid the confounding effects of cross-reacting between EBV and LCV, blood samples were test before infection, and no EBV was detected in any sample we used. Moreover, EBV-like LCV which transform B cells was also not detected during long-term culture of uninfected PBMC cells *in vitro*. In our previous study, we used *in vivo* assays to determine the suitability of the tree shrew as an animal model for EBV infection (17). In the study, we evaluated the expression of EBV genes and proteins, but further research was required to assess the reliability of the tree shrew model for EBV infection.

**Figure 1:**
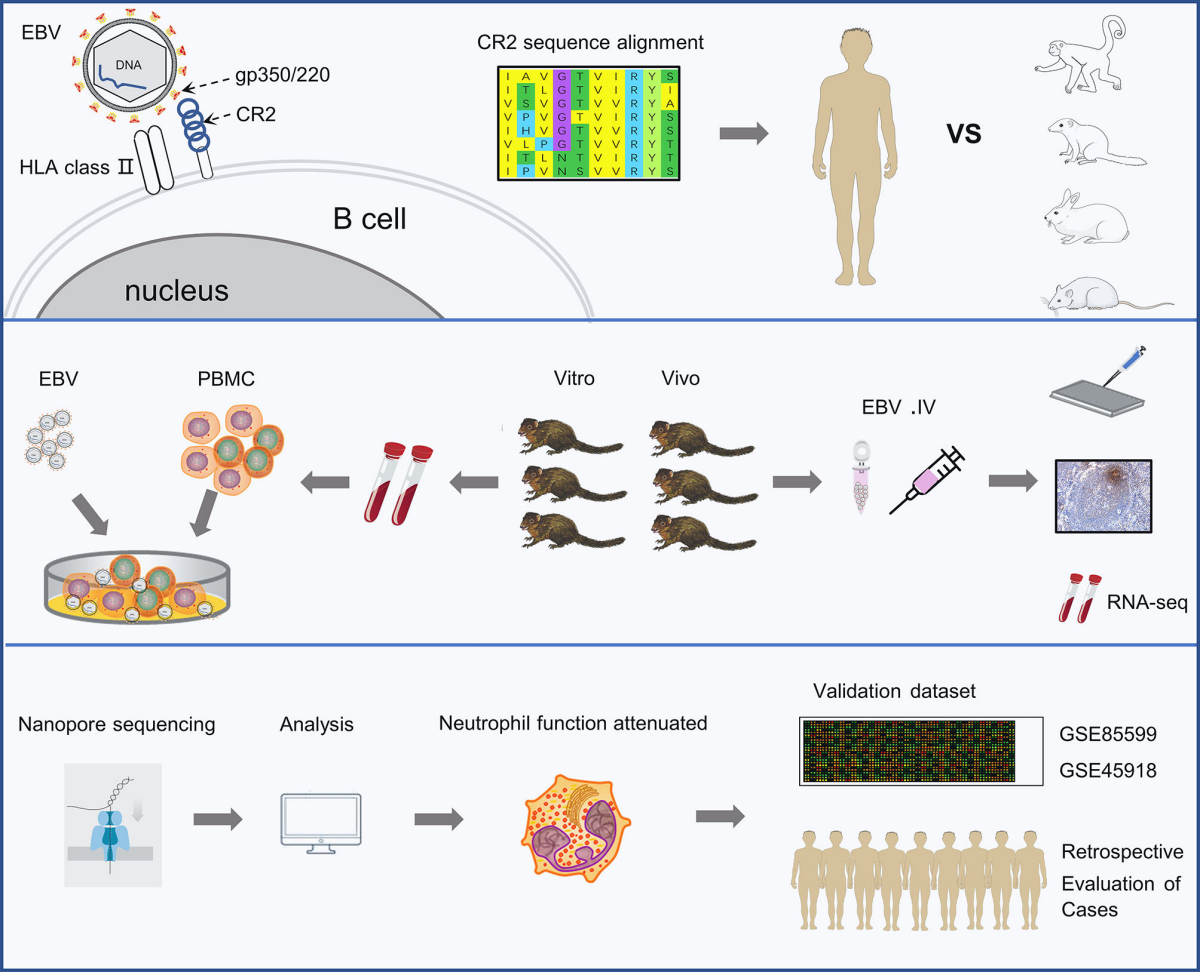
Schematic representation of the study. The experiments were completed in 3 phases. The first is identifying the key residues in the CR2 receptor that bind the gp350 protein and facilitate viral entry. The second is analyzing the characters of EBV infection in tree shrews both *in vitro* and *vivo*. The last phase was revealing the dynamic changes in biological processes occurring during EBV primary infection by RNA sequencing and validating findings using two external validation set and retrospective case reviews.

In this study, we first compared the potential species-specific residues in CR2 protein that contribute to EBV entry between tree shrew and five other species. Thereafter, we characterized the tree shrew model for EBV infection using *in vivo* and *in vitro* assays. In addition, we carried out RNA sequencing (RNA-seq) of blood samples to understand the dynamic changes in biological processes during the EBV primary infection *in vivo*. Finally, we explored the role of neutrophils in EBV primary infection in the tree shrew model and validated the findings using GEO datasets and retrospective case reviews (**Figure 1**).

## Results

### Comparative Analysis Identified the Potential Residues That Contribute to EBV Entry in Different Species

**Figure 2:**
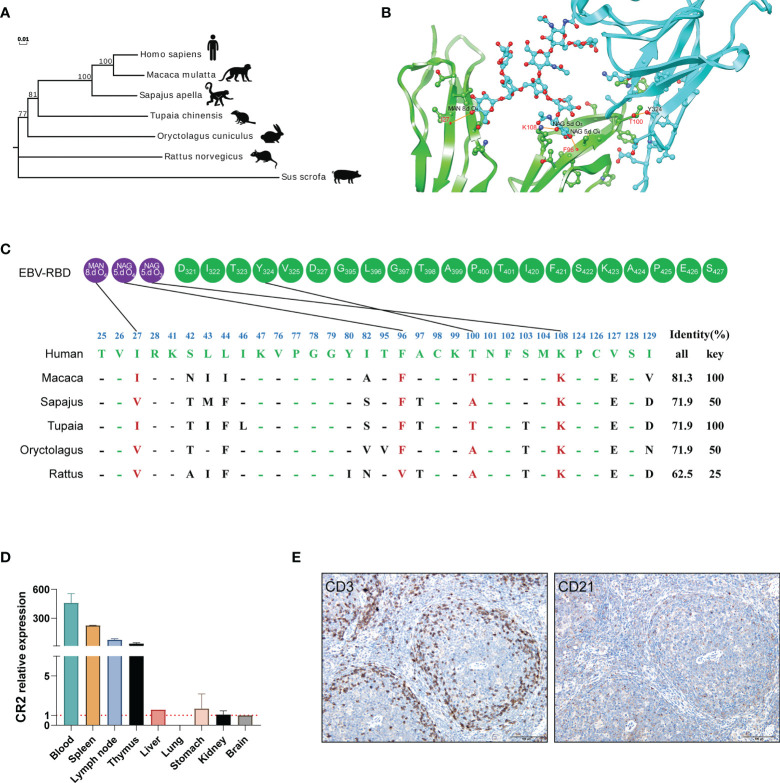
The potential residues in CR2 that restrict EBV entry. **(A)** A phylogenetic tree (constructed using the Neighbor-joining method in program MEGA7) based on the protein sequences of CR2 orthologs. **(B)** Cartoon of the binding interface between human CR2 and the EBV receptor-binding domain (RBD). CR2 and the EBV RBD are colored in green and azure, respectively. The interaction among key residues (I27, F96, K108, and T100) are shown as ball-and-stick representations. **(C)** Alignment of the residues of humans, Macaca, Sapajus, Tupaia, Oryctolagus, and Rattus SCR1-SCR2(two CR2 repeats regions where EBV glycoprotein gp350/220 attaches) at the interface of SCR1-SCR2 with the EBV-gp350. Purple circles represent the viral glycoproteins and Green circles represent the viral amino acid residues. MAN and NAG represent binding site for the glycoprotein residue, respectively. 8.d O4, 5.d O4 and 5.d O3 represent the specifics locations of oxygen atoms which form hydrogen bonds. The restrictive residues of the Macaca, Sapajus, Tupaia, Oryctolagus, and Rattus SCR1-SCR2 are highlighted in red. Favorable residues of human SCR1-SCR2 are highlighted in green. “All” represents the sequence identity of SCR1-SCR2 between humans and the other species, whereas “Key” represents the sequence identity of key residues between humans and the other species. **(D)** RT-qPCR detection of CR2 RNA in the indicated tissues from healthy tree shrews, brain tissue served as a negative control. **(E)** IHC detection of CD3 and CD21 proteins of the spleen of healthy tree shrew, positive proteins are shown in brown.

The interaction of a virus with a species-specific receptor is a primary genetic determinant of host tropism and therefore constitutes a major interspecies barrier at the level of viral entry (18–20). CR2 (complement C3d receptor 2) is expressed on the surface of B lymphocytes, and its binding to Epstein-Barr virus is a prerequisite for viral infectivity (21–23). SCR1 and SCR2 are the two most membrane distal CR2 repeats where EBV glycoprotein gp350/220 attaches (24, 25). To ascertain whether the genetic determinants would restrict the viral receptor affinity for tree shrews CR2, we analyzed the orthologs of CR2 and SCR1-SCR2, particularly the residues of the EBV receptor-binding domain (RBD), across a diverse set of species (**Figure 2C**). Phylogenetic analysis revealed that tree shrew was genetically closer to primates than rodents, based on the CR2 (**Figure 2A** and **Supplementary Table 1**) and the SCR1-SCR2 protein sequences (**Supplementary Table 1**). Through molecular modeling for human SCR1-SCR2 complexed with the EBV RBD, we found 32 potential binding sites on the receptor. Out of these sites, we identified 4 key residues that formed hydrogen bonds and provided a substantial amount of energy to stabilize the CR2-gp350 complex (**Figures 2B, C** **)**. Of note, the key residues of tree shrew shared 100% identity with human, which was much higher than Sapajus apella (50%), Oryctolagus cuniculus (50%), and Rattus norvegicus (25%) (**Figure 2C**). Finally, we compared the distribution of CR2 in 9 different tree shrew tissues using RT-qPCR. High expression of CR2 was observed in the spleen, lymph node, and thymus, with limited expression in other tissues (**Figure 2D**). Moreover, results of immunohistochemical analysis indicated that CD21 (CR2)-positive cells were mainly distributed in the germinal center, while CD3 staining was largely absent in the germinal centers of the spleen (**Figure 2E**). Overall, these results indicate that tree shrew may be an ideal animal model for studying EBV infection.

### Susceptibility of Tree Shrew Primary Cells to EBV Infection

**Figure 3:**
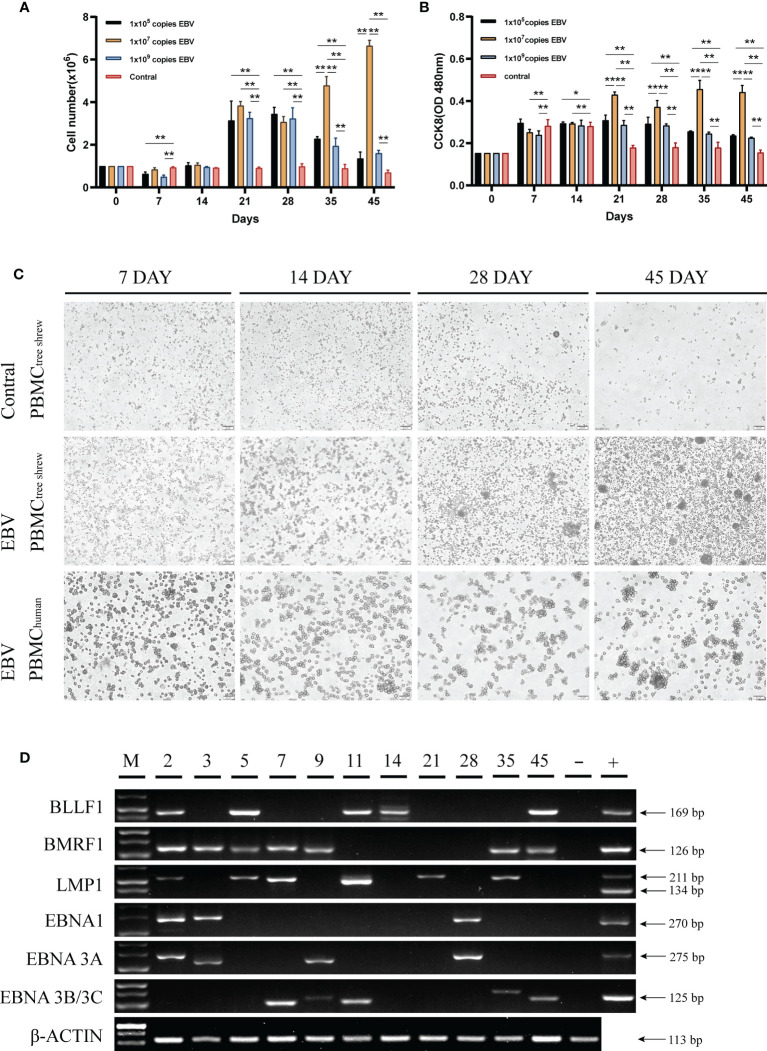
Susceptibility of tree shrew primary cells to EBV infection. **(A)** Growth levels of PBMCs, measured by cell counting. **(B)** CCK8 assay assessment of the proliferation of PBMCs after treatment with different copy numbers of EBV. **(C)** Changes in PBMCs morphology of tree shrew and human, detected by light microscopy at 7 dpi, 14 dpi, 28dpi, 45 dpi after treatment with 1x10^7^ copies EBV. Control cells were treated with an equal volume of 1640 medium. **(D)** Agarose gel electrophoresis of the EBV encoded genes from PBMCs treated with 1x10^7^ copies EBV for 49 days by RT-PCR. *p < 0.05, **p < 0.01.

Human peripheral blood mononuclear cells (PBMCs) are susceptible to EBV infection and EBV promotes proliferation and immortalization of infected cells *in vitro* (26, 27). To determine if tree shrew PBMCs had similar characteristics, we first examined the effect of different virus concentrations on the proliferation of tree shrew PBMCs. As shown in **Figure 3A**, a significant increase in the number of tree shrew PBMCs was observed (P < 0.01 compared to control group) after 21 days of co-culture with 1x10^7^ copies of EBV. The number of PBMCs in this group showed continuous proliferation throughout the observation period, while the number of PBMCs in the other two groups decreased significantly after day 28. A similar trend was observed using the CCK8 assay indicating that increase in cell proliferation was consistent with the increase in cell numbers (**Figure 3B**). Therefore, 1x10^7^ copies of EBV were used for subsequent assays. Cell morphological analysis clearly showed that PBMCs co-cultured with EBV had a tendency to grow in clusters after 14 days and these clusters gradually formed compact round-like multicellular aggregates over time **(** **Figure 3C** **).** However, apoptosis was induced in control cells cultured without EBV. To further verify that tree shrew PBMCs are indeed susceptible to EBV infection, we detected the expression of EBV genes (BLLF1, BMRF1, LMP1, EBNA1, EBNA3A, and EBNA3B/3C) in the tree shrew PBMCs throughout the co-culture period (**Figure 3D**). These results suggest that tree shrew PBMCs were susceptible to EBV infection.

### EBV Infection Caused Fever and Weight Loss in Tree Shrew

**Figure 4:**
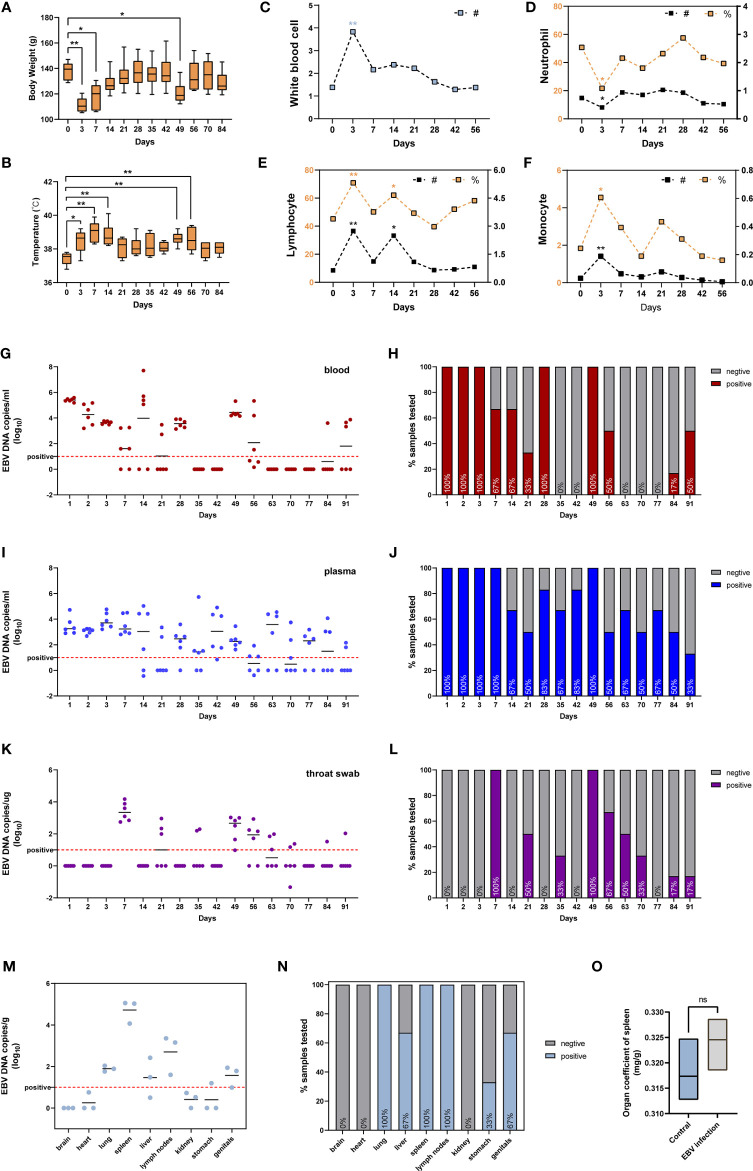
Characterization of tree shrews with EBV infection *in vivo*. **(A)** The body weight and **(B)** rectal body temperatures changes, presented as mean and standard deviation (SD). Routine blood test, including **(C)** white blood cell, **(D)** neutrophil, **(E)** lymphocyte, and **(F)** monocyte presented as mean; # represents absolute count (10^9^/L), % represents a percentage (%). The kinetics and time courses of the viral loads in **(G, H)** blood, **(I, J)** plasma, and **(K, L)** saliva samples, determined by qPCR. The detection limit is indicated by the dotted line. **(M, N)** qPCR detection of viral loads in different tissues of EBV infected tree shrews at 91 dpi. **(O)** Organ coefficient of the spleen in EBV infected tree shrews and control groups at 91 dpi. *p < 0.05, **p < 0.01, ns P > 0.05.

We also assessed the susceptibility of adult tree shrews to EBV infection. Six tree shrews were inoculated with 200ul (1x10^8^ copies/ml) EBV suspensions *via* the femoral vein injection. The body weight (BW) of each animal was measured using an electronic scale before inoculation (day0) and after infection at various time points (day 3, day 7, day 14, day 21, day 28, day 35, day 42, day 49, day 56, day 70, day 84) to monitor BW changes during the whole experimental period. **Figure 4A** shows that the BW initially decreased and then increased over time. Specifically, inoculation of tree shrews with EBV caused significant weight loss at 3 days and 7 days post-inoculation (dpi) (p < 0.01 to 0 dpi). However, the lowest BW was recorded at 3 dpi, after which the BW gradually recovered until14 dpi when the weights were indistinguishable from 0 dpi (p > 0.05 to 0 dpi, **Figure 4A**). Interestingly, loss of BW was also observed at 49 dpi (p <0.05 to 0 dpi), probably due to viral resurgence in tree shrews which will be described in detail later. The rectal temperature (RT) was also taken simultaneously with the BW measurement, using a mercury thermometer. The results indicated that RT began to rise after inoculation and reached the maximum mean temperature on 7 dpi (39.03 ± 0.62°C, p < 0.01 to 0 dpi), after which it gradually returned to normal (0 dpi, 37.43 ± 0.37°C) (**Figure 4B**). An increase in RT was also observed at 49 dpi, corresponding to the time of BW loss mentioned earlier. Remarkably, there were no mortalities throughout the experiment, although most tree shrews presented with symptoms such as fever and weight loss, while other clinical signs such as reduced activity, increasing weakness, slight diarrhea and wet-tail were observed in a few animals. These features might be a part of Infectious Monocytosis (IM) which is considered to be a self-limited infection caused by primary EBV infection (28, 29).

### Routine Blood Tests

The WBC (white blood cell) count and the main immune cell subsets (lymphocytes, neutrophils, monocytes) counts and percentages in the peripheral blood of tree shrews were measured at 0 dpi, 3 dpi, 7 dpi, 14 dpi, 21 dpi, 28 dpi, 42 dpi, and 56 dpi. The WBC count was significantly elevated at 3 dpi (**Figure 4C**, p < 0.01 to 0 dpi). At the same time, we also observed an increase in total cell counts and percentages of lymphocytes (**Figure 4E**, p < 0.01 to 0 dpi) and monocytes (**Figure 4F**, p < 0.05 to 0 dpi). Unexpectedly, a decline in neutrophils levels, the first line innate immune defense for the host against microbes and viruses (30, 31), was observed (**Figure 4C**, p < 0.05 to 0 dpi). The immuno-suppressive effect of EBV is one of the mechanisms of evading immune surveillance to establish life-long infection in the host (32, 33). Later in the manuscript, we investigated if EBV evades immune surveillance by dampening the functions of neutrophils.

### Viral DNA Loads in Blood, Plasma and Throat Swab After EBV Infection

To further characterize the dynamics of EBV infection *in vivo*, blood, plasma and throat swab samples were collected from tree shrews throughout the experimental period and subjected to virological assays. EBV viremia was readily detected in blood and plasma samples of all the animals at 1-3 dpi and 49 dpi after femoral vein infection (**Figures 4H, J** **)**. The peak viral load of blood samples appeared at 1 dpi and the viral loads ranged from 10^5.19^ to 10^5.59^ copies/ml (**Figure 4G**), however the peak of plasma samples ranged from 10^2.90^ to 10^4.73^ copies/mL at 3dpi (**Figure 4I**). In addition, EBV viremia was observed in all animals for the first 3 consecutive days after inoculation, but thereafter viremia was only observed in some animals during the experimental period of 91 days (**Figures 4H, J** **)**. These results were in line with the characteristics of EBV that can lurk in the human body for a long time after infection without any symptoms and thereafter be activated to cause recurrent infections (3, 34). Interestingly, the EBV DNA in throat swab was significantly delayed with respect to the viremia kinetics, and was only detectable at 7 dpi and 49 dpi in most animals (100%). The mean viral loads and duration in throat swabs were appreciably lower than those in blood and plasma (**Figures 4K, L** **)**.

### Tissue Distribution of EBV Infection in Tree Shrew

To determine the tissue tropism of EBV in tree shrews, six animals (three untreated and three treated with EBV) were sacrificed at 91 dpi. Quantitative PCR (qPCR) analysis showed the presence of EBV DNA in multiple peripheral organs, including the lung, liver, spleen, lymph nodes, stomach and genitals (**Figure 4N**). Similar to EBV infection in humans, the immune organs (spleen and lymph nodes) had higher loads and positivity rate of the virus (**Figures 4M, N** **)** (35). No viral DNA was detected in untreated control animals. Interestingly, there was no significant difference between the control animals and EBV infected animals with respect to the organ coefficient of spleen (**Figure 4O**). This suggested that tree shrews could carry long-lasting, asymptomatic EBV infection.

**Figure 5:**
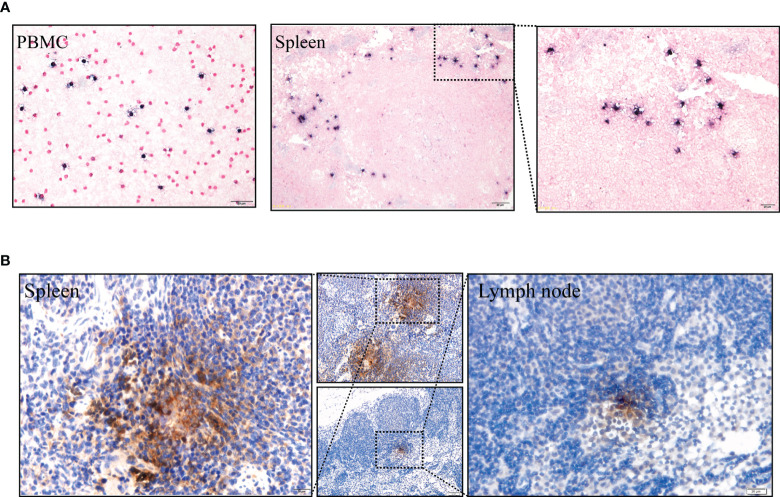
**(A)** EBERs expression of PBMCs and spleen, detected by *in situ* hybridization at 91 dpi. Positive staining is gray/black, nuclei counterstained with neutral red. **(B)** Immunohistochemical detection of LMP1 protein of the spleen and lymph node at 91 dpi. Positive viral proteins and negative cells are shown in brown and blue, respectively.

Moreover, LMP1 protein was detected in the spleen and lymph nodes using immunohistochemistry (**Figure 5B**, positive and negative controls pictures in **Supplementary Figure 3**). To further confirm the presence of EBV, Epstein Barr virus-encoded RNA (EBER) was detected using *in situ* hybridization studies. Clear EBER-positive puncta were observed in PBMCs and spleen (**Figure 5A**, positive and negative controls pictures in **Supplementary Figure 3**). Similar results were observed in all experimental animals after being sacrificed.

### Time Course Transcriptomics Revealed Genes and Biologic Processes Important for Infection

**Figure 6:**
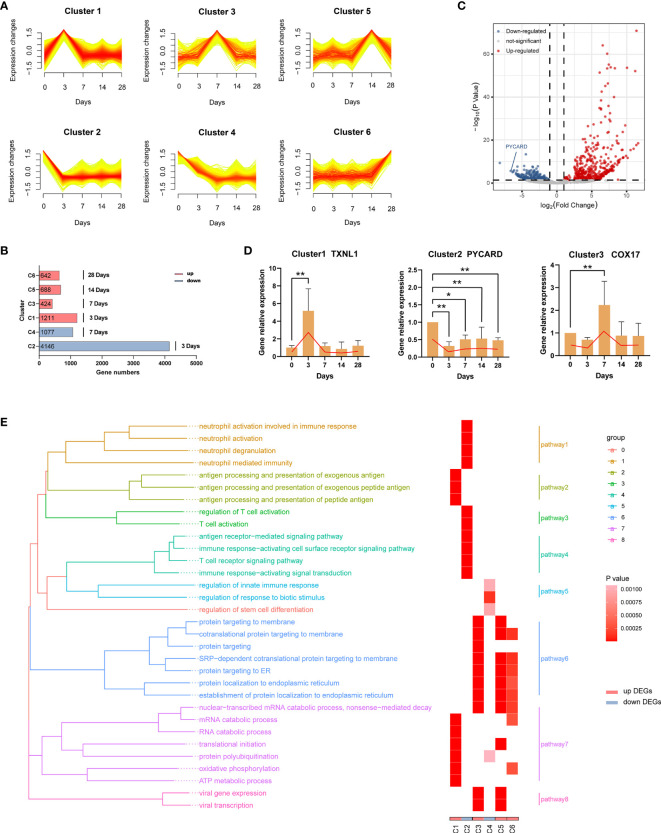
Dynamic transcriptome changes induced by EBV infection. **(A)** Mfuzz clustering identified six distinct temporal patterns of genes expression according to expression profiles. The X-axis represents five-time points, while the Y-axis represents log2-transformed, normalized intensity ratios in each time point. **(B)** The number of genes in each cluster. Genes in each cluster were classified as characteristic genes at four time points after EBV infection according to expression pattern. **(C)** The volcano plot of 3 dpi vs 0 dpi DEGs. **(D)** Validation of RNA-Seq results using RT-qPCR (TXNL1, PYCARD, COX17). Twelve genes (two genes per cluster) are shown in **Supplementary Figure 2**. **(E)** Gene Ontology (GO) enrichment analysis of DEGs in each cluster; GO categories were collapsed into 8 pathways according to GO-term similarities by GOSemSim package. *p < 0.05, **p < 0.01.

To better understand the biological processes of the EBV primary infection *in vivo*, we performed RNA sequencing (RNA-seq) of blood samples at five distinct time points (0 dpi, 3 dpi, 7 dpi, 14 dpi, 28 dpi, three replicates per time point). First, we used Mfuzz (36) to cluster genes with similar temporal expression profiles. Results of cluster analysis showed that there were six types of expression patterns including; initial increase in gene expression that peaked at 3 dpi (Cluster 1); initial decline in expression that reached its lowest at 3 dpi (Cluster 2); increase in expression from 3 dpi that peaked at 7 dpi (Cluster 3); decline in expression from 3 dpi that reached its lowest at 7 dpi (Cluster 4); increase in expression from 7 dpi that peaked at 14 dpi (Cluster 5); and increase in expression from 14 dpi that peaked at 28 dpi (Cluster 6) (**Figure 6A**). The increase indicated up-regulation, while the decline indicated down-regulation, and the greater the change in gene expression, the more important the genes. Hence, we considered the genes in the six clusters as the characteristic genes for each time point depending on when maximum or minimum expression was observed. Specifically, genes in cluster 1 (C1) were the characteristic up-regulated genes at 3 dpi, genes in cluster 2 (C2) were the characteristic down-regulated genes at 3 dpi, genes in cluster 3 (C3) were the characteristic up-regulated genes at 7 dpi, genes in cluster 4 (C4) were the characteristic down-regulated genes at 7 dpi, genes in cluster 5 (C5) were the characteristic up-regulated genes at 14 dpi, while genes in cluster 6 (C6) were the characteristic up-regulated genes at 28 dpi, as shown in **Figure 6B**. Then we used DESeq2 (37) to identify the differentially expressed genes (DEGs) between each time point after infection and 0 dpi (**Supplementary Table 3**). A total of 879 DEGs between 3 dpi and 0 dpi were identified, including 524 up-regulated genes (i.e., C1) and 355 down-regulated genes (i.e., C2) **(** **Figure 6C** **)**. **Figure 6D** shows the validation results of the DEGs (TXNL1, PYCARD, COX17) using RT-qPCR. A total of 12 genes (two genes per cluster) are shown in **Supplementary Figure 2**.

In order to explore the biological functions of the DEGs, we performed Gene Ontology (GO) enrichment analyses (**Supplementary Table 4**). After filtering, 32 GO categories were further collapsed into 8 pathways (**Figure 6E**) using the GOSemSim package (38). At 3 dpi, pathways involving antigen processing and presentation (C1, pathway2) as well as nucleic acid, protein and energy metabolism (C1, pathway7) were enriched in response to EBV infection. Interestingly, the immune functions of neutrophils and T cells were suppressed over the same period (C2, pathway 1, 3 and 4). On the one hand, a large number of proteins were processed to maturity and transported to participate in the defense responses (C3, pathway 6) at 7 dpi, but immune suppression still persisted (C4, pathway 3). At 14 dpi and 28 dpi, similar biological processes were observed including active processing and transport of proteins (C5 and C6, pathway 6 and 7). In addition, viral transcription-related pathways were enriched at 7 dpi and 14 dpi (C3 and C5, pathway 8).

### EBV Infection Suppressed Neutrophils

**Figure 7:**
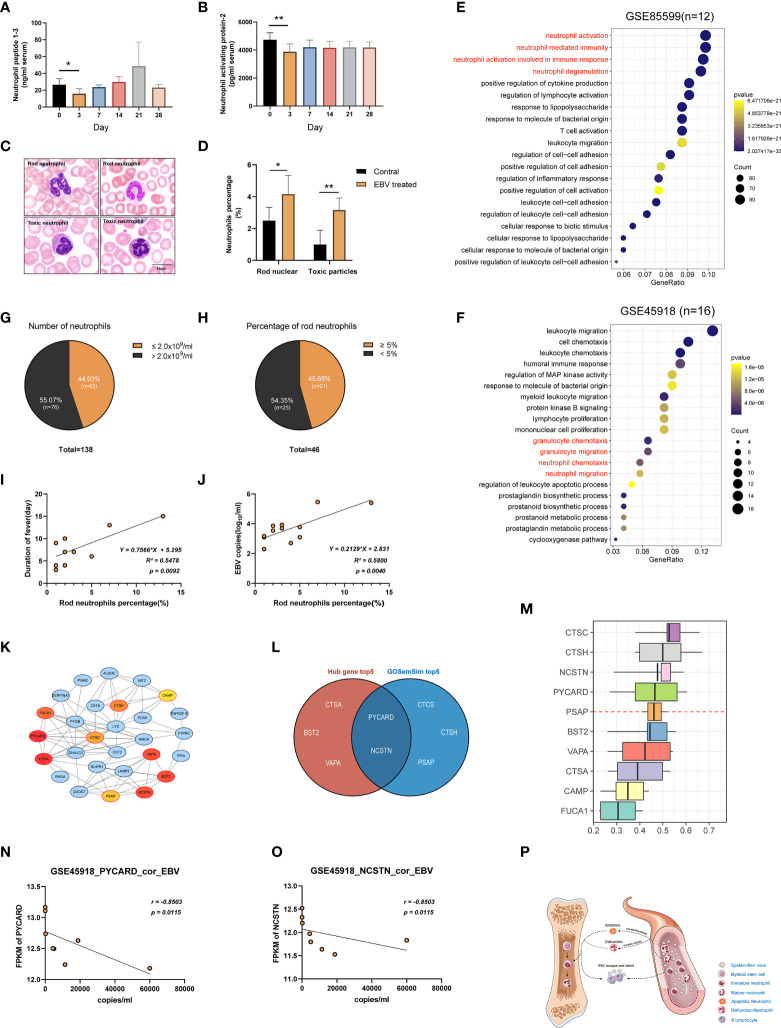
Suppression of neutrophils after EBV infection. **(A, B)** ELISA examination of Neutrophil peptide 1-3 and Neutrophil activating protein-2 were examined. **(C)** Morphology of neutrophils with rod-shaped nucleus or specific intoxication particles in the cytoplasm in the blood of tree shrews (Wright-Giemsa stain). **(D)** The ratio of neutrophils with a rod-shaped nucleus or specific intoxication particles in peripheral blood smear. **(E, F)** GSE85599 and GSE45918 datasets of patients with infectious mononucleosis (IM) demonstrating the function of neutrophils was downregulated based on GO enrichment analysis of down-regulated genes in the datasets. **(G, H)** The proportion of patients with a reduced number of neutrophils and an increased percentage of rod-shaped nucleus neutrophils in the reviewed cases with IM. **(I, J)** A simple linear regression model between the percentage of rod-shaped nuclear neutrophils, days of fever, and EBV DNA copies of blood was generated to determine R^2^ (coefficient of determination) and P-value (Significance of the correlation). **(K)** The PPI network of the neutrophil-related pathway in tree shrews at 3 dpi. Node color reflects its DMNC scores, such that a higher score of a node is denoted by a redder color of the node. The nodes with red borders represent the hub genes. **(M)** The weight value of the top 10 genes according to GO semantic similarity. The horizontal axis refers to the value of weight. **(L)** The Venn diagram showing the intersection of top5 genes of DMNC score and top5 genes of the weight value of GO semantic similarity. **(N, O)** Correlations of gene expression levels of PYCARD and NCSTN with EBV copy number in GSE45918 dataset. r-values represent the Spearman correlation coefficient. **(P)** Neutrophil suppression hypothesis. *p < 0.05, **p < 0.01.

As mentioned in the preceding sections, a decrease in the number and function of neutrophils was observed in tree shrews at 3 dpi. To further confirm this phenomenon, we performed a series of experiments. First, the serum levels of Neutrophil peptide 1-3 (NP1-3) and Neutrophil activating protein-2 (NAP-2) were determined using ELISA assays in the tree shrews at 3 dpi, 7 dpi, 14 dpi, 21 dpi, and 28 dpi. The results showed a significant decrease in proteins-related to neutrophil functions at 3 dpi (**Figures 7A, B**; p < 0.05 to 0 dpi). Moreover, peripheral blood smears revealed morphological changes in neutrophils at 0 dpi and 3 dpi. As shown in **Figures 7C, D**, EBV infection induced an increase in the percentage of immature neutrophils (rod-shaped nucleus) and neutrophils which contained specific intoxication particles in the cytoplasm (p < 0.05). Two publicly available RNA-seq data (GSE85599 (39) and GSE45918 (40)) of IM from the Gene Expression Omnibus (GEO) database were used to validate these results in humans. IM is considered to be a symptom of EBV primary infection (41). Downregulated genes from the RNA-seq data were subjected to gene set enrichment analyses for GO-biological process. **Supplementary Table 5** and **Figures 7E, F** show the results of pathways enrichment analysis, clearly displaying that neutrophil activation pathways, neutrophil-mediated immune pathways, neutrophil chemotaxis pathways and neutrophil migration pathways were significantly enriched. Additionally, to further understand the relationship between neutropenia and percentage of rod nuclear neutrophils and clinical symptoms, the data of 138 inpatients with IM were retrospectively analyzed (**Supplementary Table 6**). Neutropenia (neutrophil count ≤ 2x10^9^/ml) was recorded in 44.93% (62/138) of the patients (**Figure 7G**). Among the patients with neutropenia, 74.19% (46/62) had records of peripheral blood smear examination, with 45.65% (21/46) of the samples showing increase in the percentage of rod nuclear neutrophils (**Figure 7H**, percentage ≥ 5%). This suggests that decreased neutrophil count and the increase in the percentage of rod nuclear neutrophils are commonly associated with EBV infection. Moreover, results of regression analysis revealed that there was a moderate positive correlation among percentage of rod nuclear neutrophils, days of fever (**Figure 7I**, R^2^ = 0.5478, P = 0.0092) and copies of EBV DNA in blood (**Figure 7J**, R^2^ = 0.5800, P = 0.0040). Thus, a decrease in neutrophil count as well as the increase in the percentage of rod nuclear neutrophils are potential immune escape strategies for EBV.

### The Critical Genes for Neutrophils Dysfunction After EBV Infection

To uncover the critical regulatory genes, we first extracted genes from the neutrophils function related pathways and constructed a PPI network through STRING database (https://string-db.org/). Genes with the top10 DMNC scores were identified as hub genes using Cytoscape plug-in cytoHubba (**Supplementary Table 7**). As shown in **Figure 7K**, the darker the red color, the higher the scores. We further calculated the weight value of top10 genes according to GO semantic similarity using GOSemSim package (**Figure 7M**). Finally, critical genes (PYCARD and NCSTN) were selected by taking the intersection of the top 5 genes of DMNC scores and top 5 genes of weight value (**Figure 7L**). Moreover, it was interesting that the FPKM values of PYCARD and NCSTN in the GSE45918 data set had a strong negative correlation with the corresponding copies of EBV DNA in blood (**Figures 7N, O**, r = -0.8503, p = 0.0115).

## Discussion

EBV has a restricted host range which only includes humans and some nonhuman primates (NHP). This has resulted in the unavailability of suitable EBV animal models, which has limited the progress in EBV-related research (42, 43). In the first section, we found that restriction of EBV infection is attributed to the differences in CR2 sequence homology, especially the residues of EBV-RBD. The CR2 protein sequence homology of Tupaia chinensis (Scandentia) is higher than Oryctolagus cuniculus (Lagomorpha) and lower than Sapajus paella (Primates), which was compatible with the evolutionary genomic classification (11). More importantly, the key residues of tree shrew shared 100% identity with human. However, some of these key residues differ in Rattus norvegicus (V27, V96 and A100). It is conceivable that these substitutions disrupt the interaction of these CR2 orthologs with the viral gp350 and thus impair viral entry. These indicate that tree shrews are more suitable as the animal models of EBV infection than other non-primate species. This conclusion is further validated by the results of *in vitro* assays showing that EBV not only enters the PBMCs of tree shrews and expresses its genes and proteins, but it also increases the proliferation of the PBMCs (**Figure 3**). This is consistent with the general characteristics of the EBV infection of Human PBMCs (27).

The natural course of the EBV infection involves a dynamic and complex interplay between the virus and the immune system of the host (44). Therefore, further *in vivo* experiments were required to confirm the reliability of the tree shrew model. The route of oral mucosa infection is more similar to the natural course of infection with EBV; however, it is worth noting that the infection bases on the basis of some anatomical structures in the oral mucosa, for example: the pharyngeal lymphoid ring (45). Under the precondition that anatomical structures in the oral mucosa of tree shrews are not yet fully understood, femoral vein infection that may have more efficient and stable compared with other routes was selected to evaluate tree shrews suitability for EBV infection. The same choice can also be seen in a number of reports that indicate rabbits can be infected with EBV (46, 47).

A decrease in body weight and increase in temperature was a reflection of disease progression. Interestingly, elevated body temperature and decreased body weight at the initial stages of infection and at 49 dpi was also observed in tree shrews. In addition, the simultaneous occurrence of EBV viremia at 49 dpi was an indication of EBV resurgence (**Figure 4**). Thereafter, the weight gradually returned to normal and remained that way until the end of the experiments. There was no significant difference in the organ coefficient of spleen between the control animals and EBV infected animals at 91 dpi. This suggested that tree shrews could carry long-lasting, asymptomatic EBV infection. It should be pointed out that EBV activity could still be observed until tree shrews were sacrificed (**Figures 4M–O**, **5**). This is possibly due to the unusual infection route; immune surveillance might not be efficiently induced in the host animals that may cause for spontaneous activation of EBV as the latency II or III programs rather than maintained as latency 0 or I. The detail mechanisms will be further investigated in the future.

The human immune response against EBV does not usually result in the complete elimination of virus. Instead, a lifelong virus carrier status is established after primary infection and sometimes viral reactivation occurs (1). The infectious life cycle of the EBV is characterized by replication in the oropharyngeal mucosal and secretion to infect neighboring host cells (48). In our study, EBV DNA was detected in throat swab samples at 7 dpi and 49 dpi. However, the infectivity of the EBV in saliva samples was not evaluated because saliva samples were very difficult to collect. There is need for further study in this area. There are several reports indicating that EBV is shed from more than one mucosal site such as genitals, suggesting a potential sexual route of transmission for EBV (49, 50). Interestingly, EBV DNA was also detected in the genital of tree shrews (67%), indicating that the tree shrew model could be useful for evaluating possibility of sexual transmission of EBV. These results strongly support that tree shrews are suitable and effective small animal models of EBV infection.

To understand the dynamic immune response induced in EBV-infected tree shrews, we investigated the transcriptome changes in blood samples at 0 dpi, 3 dpi, 7 dpi, 14 dpi, and 28 dpi. RNA-seq results showed that genes for many biological processes were differentially expressed between different time points (**Supplementary Table 4**), revealing the complexity of transcriptome regulation during EBV infection. Notably, neutrophil immune function-related pathways were suppressed at 3 dpi, accompanied by a decline in the number of neutrophils as seen in routine blood examination. The suppression of neutrophils has been demonstrated in viral infections (51–53). For example, influenza virus can inhibit lysozyme secretion by neutrophils (54). However, there are only a few studies that have focused on the changes in the neutrophils after EBV infection (55, 56). In our study, neutrophil dysfunction is suggested based on data. These findings in the tree shrew model were validated by similar results from pathway enrichment analysis using two human data datasets. Moreover, there was a significant increase in the percentage of immature neutrophils (rod-shaped nucleus) in peripheral blood at 3 dpi. The immature neutrophils have impaired immune response and are considered to be immuno-suppressive. The increase in the number of immature neutrophils in peripheral blood indirectly reflects the suppression of neutrophil function (57, 58). This indicates that Primary EBV infection is indeed associated with neutrophil dysfunction. We also reviewed the clinical characteristics of IM cases and found that almost half of the patients (62/138) had neutropenia in peripheral blood. This is consistent with the findings of Carter et al. (up to 50%) (59, 60). Moreover, we also found that there was an increase in the percentage of rod nuclear neutrophils in 45.65% of the patients (21/46), and that this increase was positively correlated with prolonged length of days of fever. This suggests that the course of IM might be associated with the percentage of immature neutrophils and that this percentage can be of prognostic significance. A similar trend was observed in COVID-19 patients, whereby, a dramatic increase in the number of immature neutrophils was observed in peripheral blood and immature neutrophil-to-VD2 T-cell ratio was an early marker for severe COVID-19 (61–63). What is even more interesting is that the percentage of rod nuclear neutrophils was positively correlated with copies of EBV DNA in blood. This suggests that the decrease in neutrophil count as well as the increase in the percentage of rod nuclear neutrophils can be attributed to EBV infection. This may be a strategy by EBV to evade the host innate immune response during primary infection. A review of literature reveals that EBV can suppress several types of immune cells such as T and B lymphocytes as wells as myeloid cell such as macrophages and dendritic cells (64–67). However, there has been little focus on neutrophils. It is evident that the occurrence of transient acute neutropenia during the course of EBV infection is common in most patients suffering from IM (68–72). Interestingly, Larochelle et al. found that EBV infection drastically increases the rate of spontaneous neutrophil apoptosis *in vitro* and that Fas signaling pathway plays an essential role in the process (73). Here, we suggest a mechanism of immune escape by EBV, which we call the neutrophil suppression hypothesis (**Figure 7P**). Specifically, primary EBV infection decreases the number of neutrophils in peripheral blood directly by inducing apoptosis of neutrophils *via* the Fas signaling pathway. In addition, the infection may also indirectly downregulate neutrophil function by downregulating functional related-genes of neutrophils, such as PYCARD and NCSTN. On one hand, this weakens the first line of host defense against invading pathogens leading to evasion of the innate immune response and thus providing opportunities for EBV to establish long-term latent infection in the host. On the other hand, this stimulates hematopoiesis and increases the release of immature neutrophils from the bone marrow into the peripheral blood. While immature neutrophils increase the total number of neutrophils in peripheral blood, immature neutrophils are tolerant to virus infection, thereby helping the virus evade immune surveillance.

In summary, the genetic basis defining the host range of EBV demonstrates a definite superiority of tree shrew over other nonprimates. Further analysis through *in vivo* and *in vitro* experiments provides strong evidence that tree shrew is susceptible to EBV infection and exhibits similar characteristics to humans. Moreover, based on the tree shrew model, the decreased count and dysfunction of neutrophils were revealed, which may be a strategy for EBV to evade host innate immunity during primary infection. As such, the established novel tree shrew animal model provides an avenue to explore the pathogenesis of EBV infection and is a valuable platform for evaluating the efficacy of vaccines and therapeutic drugs.

## Material and Methods

### Phylogenetic and Identity Analysis of Epstein-Barr Virus Receptor Among Tree Shrew, *Homo sapiens*, and Other Species

The reference Epstein-Barr Virus Receptor (CR2, complement C3d receptor 2) protein sequence used for constructing the distinct phylogenetic branches and identity analysis, were obtained from the GenBank sequence database of NCBI under the following accession numbers: Homo sapiens (NP_001006659.1), Macaca mulatta (XP_014973177.2), Sapajus apella (XP_032144963.1), Tupaia chinensis (XP_014447439.1), Oryctolagus cuniculus (XP_017203311.1), Rattus norvegicus (NP_001099459.2) and Sus scrofa (XP_020918993.1). Phylogenetic analyses of the CR2 protein sequence were performed with the MEGA (version: 7.0.26). Phylogenetic trees were assembled *via* the Neighbor-Joining method of the Maximum Composite Likelihood model (74). Statistical evaluation of the branching pattern was performed by bootstrap analysis of 1,000 replicates. The EMBOSS NEEDLE online program (https://www.ebi.ac.uk/Tools/psa/emboss_needle/) was applied to reveal the identity scores among protein sequences.

Homo sapiens SCR1- SCR2 protein sequence was obtained from RSCB Protein Data Bank (PDB; http://www.rcsb.org/pdb/), PDB id: 1LY2. A global-local alignment (https://www.ebi.ac.uk/Tools/psa/emboss_needle/) between the Homo sapiens SCR1- SCR2 protein sequence and the other species CR2 segment was performed to identify the SCR1- SCR2 homologous protein segment across species (**Supplementary Data 1**). The EMBOSS NEEDLE online program was employed to determine the identity scores among protein sequences. The 3D spatial structure of the SCR1- SCR2 protein (PDB ID: SCR1-21ly2) and receptor-binding domain (RBD) of EBV (gp350) (PDB ID: SGP3502h6o) were obtained from RCSB Protein Data Bank. Molecular modeling and visualization of the protein-protein (SCR-gp350) interactions were performed using the Chimera software (http://www.cgl.ucsf.edu/chimera, in the public domain). Two residues were defined to be in contact if the distance between any of the atom pairs was less than 5Å. Homo sapiens SCR1-2 residues having contact with gp350 were screened by executing the command “select:.#1 &:.#0 zr < 5” and “select:.#0 &:.#1 zr < 5”. Subsequently, using the EMBOSS NEEDLE online program, the Homo sapiens residues were compared to those of corresponding residues in other species. Lastly, water molecules were removed and hydrogen-bond interactions for these residues were explored using Chimera software.

### Culture of Primary Tree Shrew Peripheral Blood Mononuclear Cells and EBV Infection

EBV viral supernatant was obtained from the B95-8 strain (NCBI: txid 10377) known to release high titers of transforming EBV. All experiments involving infectious EBV were conducted in biosafety level 2 (BSL2) facilities. B95–8 strain was cultured in 1640 Medium supplemented with 10% FBS and 1×antibiotics (100 kU/l penicillin, 100 mg/l streptomycin) at 37°C and 5% CO_2_ in a humidified incubator for 14 days. Next, the whole culture was transferred to a 50 mL centrifuge tube and freeze-thawed thrice to lyse cells and release the virus. The culture was centrifuged at a low speed (1200xg) to remove the cell debris. The supernatant was filtered *via* a 0.45-μm centrifugal filter device. The filtrate fluid was centrifuged at high speed (16,000 × g for 90 min at 4 °C) and resuspended in 1ml of 1640 Medium to acquire the viral supernatant for analysis. The viral supernatant was stored at −80°C until tree shrew infection (75). DNA was extracted from 200µl viral supernatant to determine viral genome copy numbers before use.

PBMCs were isolated from whole blood by Ficoll (Solarbio, Beijing, China) gradient centrifugation following manufacturer guidelines. PBMCs (1×10^6^ cells) were inoculated on 24-well plates and cultured under the same conditions as the B95-8 strain. PBMCs were assigned to three experimental groups and one control group, with three replicates per group. Subsequently, 1×10^5^ copies, 1×10^7^ copies, and 1×10^9^ copies of EBV were added in the three experimental groups, respectively, and to the control group with the same amount of 1640 medium. Following EBV infection, cell proliferation on Day 7, Day 14, Day 21, Day 28, Day 35, and Day 45 was evaluated using the CCK-8 kit (Solarbio, Beijing, China). Next, 10 µl of CCK8 solution was added to each well, and cells were incubated at 37°C for another 5 hours. The optical density was read at 480 nm by a microplate reader. The number of viable cells was manually counted under a microscope using a hemocytometer at the same time points as CCK8. According to the optimal EBV infection concentration determined above, vitro cultures were further prepared, and cell morphology was examined under a light microscope on Day 7, Day 14, Day 28, and Day 45. Human PBMCs were acquired from healthy volunteers [EBV DNA-negative, from another clinic study by our team, Ethical Approval No: 2020 (KY-E-135)] served as positive controls for better assessment of morphological changes. Total RNAs of PBMCs were extracted with the TRNzol Universal Reagent Kit (Tiangen, Beijing, China) according to the manufacturer’s protocol. RNA quantity and purity were determined using a NanoDrop2000 spectrophotometer (Thermo Fisher Scientific, USA).

### Animal Experiments and Ethics Statement

Six tree shrews (F1 generation, Production approval number: SCXK (Dian) 2020–0004, 8 ± 2 months, male and female unlimited, 138.1 ± 7.2 g) were acquired from the Kunming Institute of Zoology, Chinese Academy of Sciences. The protocols used in this study were approved by the Animal Ethics Review Committee of Guangxi Medical University (approval number: 202005015). The experimental protocols strictly followed the ‘Guiding Principles for the Use and Care of Experimental Animals issued by the Ministry of Science and Technology of China. The total number of animals and their suffering was minimized according to the 3R principle.

Six tree shrews were injected with 200ul EBV suspensions (1x10^8^ copies/ml) *via* the femoral vein as described previously. Samples were isolated the day before inoculation as 0 dpi. Blood and throat swab samples were collected on the 0 dpi, 1 dpi,2 dpi,3 dpi,7 dpi, 14 dpi, 21 dpi, 28 dpi, 35 dpi,42 dpi, 49 dpi, 56 dpi, 63 dpi, 70 dpi, 77 dpi, 84 dpi, and 91 dpi (femoral vein blood, 1 ml each time). Serum and plasma were immediately separated through centrifugation (3,000 g at 4°C) and stored at −80°C. No EBV was detected in any samples at 0 dpi. Genomic DNA was extracted from 150 µl of blood samples using the QIAamp DNeasy Blood & Tissue kit (Qiagen, Hilden, Germany) and eluted in 30 µl of nuclease-free water. Total RNA was extracted from 200 µl of blood samples with the RNAprep Pure Hi-Blood Kit (Tiangen, Beijing, China). Viral nucleic acid was extracted from 150 µl of tree shrew plasma with the QIAamp MinElute Virus Spin Kit and eluted in 20 µl of nuclease-free water. Genomic DNA of throat swab samples was extracted using TIANamp Swab DNA Kit (Tiangen, Beijing, China). The quantity and purity of the extracted nucleic acid were determined by a NanoDrop2000 spectrophotometer (Thermo Fisher Scientific, USA). Serum samples were used to detect NP1-3 and NAP-2 levels using an ELISA kit (Cusabio, Wuhan, China) following the manufacturer’s guidelines.

Three tree shrews were sacrificed at 91 dpi with an overdose of sodium pentobarbital. Subsequently, multiple tissues, including the lung, liver, spleen, lymph nodes, stomach, and genitals were harvested. Genomic DNA was extracted from 20mg of each tissue using the QIAamp DNeasy Blood & Tissue kit (Qiagen, Hilden, Germany). The remaining tissue was fixed, dehydrated, paraffin-embedded, and sectioned. For the control experiment, tissues were obtained from three healthy tree shrews. Total RNA was extracted from 20mg of the control tissues (lung, liver, spleen, lymph nodes, thymus, stomach, kidney, and brain) for CR2 detection with the RNeasy Protect Mini Kit (Qiagen, Hilden, Germany) and eluted in 60 µl of nuclease-free water.

### Quantitative Real-Time PCR (qPCR) Detection of EBV Copy Number

EBV DNA copies of viral supernatant, blood, plasm, throat swab, and tissue samples were quantified by qPCR using primers (forward primer, 5′- TCT TAG GAG CTG TCC GAG GG -3′; reverse primer 5′- CCC AAC ACT CCA CCA CAC C -3′) and a probe that targets the EBV BamHI W region (probe, 5′- CAC ACA CTA CAC ACA CCC ACC CGT CTC -3′) (76). Standard curves were generated from genomic DNA of Namalwa cell line (containing two integrated viral genomes/cell) (77, 78), purchased from the Chinese Academy of Sciences Institute of Cell Resource Center (Shanghai, China). The culture conditions for the Namalwa cells and the DNA extraction protocol were as previously described. For PCR, Takara Premix ExTaq™ (Probe qPCR) was used with manufacturer-recommended reaction conditions. All experiments were repeated 3 times. The virus load of samples was determined according to the appropriate standard curve (PCR amplification efficiency (E) of 95–105% and correlation coefficient (R^2^) > 0.995) by the Bio-Rad CFX96 detection system.

### RT-PCR Detection of EBV-Encoded Gene Expression in PBMCs

RNA (800 ng) was reverse transcribed to cDNA using the PrimeScript™ RT reagent Kit with gDNA (Takara, Dalian, China). RT-PCR was performed with TaKaRa Ex Taq^®^ (Takara, Dalian, China) according to the manufacturer’s protocol. RT-PCR detection of the expression of BLLF1, BMRF1, LMP1, EBNA1, EBNA3A, EBNA3B/3C was determined using primer sequences presented in **Supplemental Table 2**. The “housekeeping gene” β-actin was amplified as an internal control. cDNA from B95–8 cells served as a positive control. Lastly, 3% agarose gel electrophoresis (120 V for 32 min) was performed to visualize the bands under UV light. All the experiments were repeated three times.

### EBER-*In Situ* Hybridization for EBV Detection in Tree Shrew Blood and Tissues

Sequences of Epstein-Barr virus-encoded small RNA (EBER1 and EBER2) probes were acquired from the literature (79, 80). Highly sensitive digoxigenin-labeled oligonucleotide probes were synthesized by Shanghai Sangon Biotechnology Co., Ltd (Shanghai, China). Briefly, tissue sections (thick 5 um) were dewaxed regularly and digested for 30 min by pepsin (2 min of PBMCs smear) to expose RNA. Pre-hybridization was performed for 2-4 hours in a wet box with an aqueous solution of 20% glycerin to reduce the nonspecific reaction. The working concentration of the probe was 4μg/ml. A denaturation step was performed at 95°C for 15min. Subsequently, a hybridization reaction was performed using the Enhanced Sensitive ISH Detection kit (BOSTER, Wuhan, China). Nuclear fast red (Solaibao, Beijing, China) served as a chromogen. B95-8 cells were used as a positive control.

### Immunohistochemical Examination of Tree Shrews

Antibodies used for immunohistochemical experiments are listed in **Supplemental Table 8**. Blood was drawn from the femoral vein and cardiac of the animals before they were sacrificed and PBMCs isolation was performed as previously described. PBMCs were used to make a cell smear using slides treated with 0.1% gelatin/0.1% chromium potassium sulfate for improved cell attachment Tissue samples of spleen and lymph nodes were prepared and subjected to immunohistochemical analysis using SP-HRP kits (SP-9000, ZSGB Biotechnology Co. Ltd., Beijing, China) following the manufacturer’s instructions.

### RNA-seq Analysis

Blood samples harvested at 0, 3, 7, 14, and 28 dpi (three replicates per group) were used for global transcriptome analysis. Oxford Nanopore Technologies (ONT) sequencing is emerging as a new generation sequencing technology based on nanopore single-molecule real-time electrical signal (81). In most cases, the NGS RNA-Seq technology cannot assemble the entire transcripts and recognize an Isoform (transcript of homologous gene, superfamily gene, and allele expression), making it challenging for us to understand life processes. Full-length transcriptome sequencing based on ONT does not require RNA breakage; rather, RNA is directly reverse transcribed to obtain full-length cDNA. The experimental workflow is shown in web extra (https://nanoporetech.com/). The original data format of the Nanopore sequencing output data is the second-generation fast5 format containing all original sequencing signals. Base-calling was performed with the Guppy software in the MinKNOW2.2 package and data in fast5 format was converted to fastq format for subsequent quality control analysis. Short sequences (length<500bp) and low-quality reads (Qscore<6) of raw fastq data were filtered. Notably, if both ends of reads were identified as primers, they were determined as full-length sequences according to the cDNA sequencing principle. The full-length sequences statistics are shown in **Supplemental Table 9**. Consistent sequences of each sample were aligned with the reference genome (http://www.treeshrewdb.org/) by minimap2. Then, we filtered out sequences with identity lower than 0.9 and coverage lower than 0.85 to remove redundant for alignment result. CPM (counts per million) (82) served as an indicator to measure gene expression level. Differential expression analysis was performed by DESeq2 (37) with a fold change cutoff of 1 and a p-value cutoff of 0.05.

### Retrospective Case Review

The Ethics Committee of the First Affiliated Hospital of Guangxi Medical University approved this study [Approval number: 2021(KY-E-112)]. A total of 138 cases of IM which recorded the count of neutrophils were identified following a search of the First Affiliated Hospital of Guangxi Medical University case archives from 2015 to 2020.

### Statistical Analysis

All statistical data were analyzed with GraphPad Prism Software version 9.0.0 (GraphPad Software Inc., La Jolla, CA). Outliers were first detected and removed using the “ROUT” method to clean the data. The Shapiro-Wilk normality test was performed on clean data to assess for normality. For normally distributed data, comparison of two groups was performed *via* Unpaired Student t-test, whereas the Mann–Whitney U-test was applied when data did not conform to normal distribution. Comparison of more than two groups was executed using an ordinary ANOVA test with Tukey’s *post hoc* test when data were normally distributed with equal variance. However, for data that did not conform to normal distribution or variance was significantly different between groups, non-parametric tests were applied. P < 0.05 denoted statistical significance.

## Data Availability Statement

The datasets presented in this study can be found in online repositories. The names of the repository/repositories and accession number(s) can be found below: https://www.ncbi.nlm.nih.gov/, accession ID: PRJNA767811.

## Ethics Statement

The studies involving human participants were reviewed and approved by the first affiliated Hospital of Guangxi Medical University Ethical Review Committee [2020 (KY-E-135) 2021 (KY-E-112)]. The patients/participants provided their written informed consent to participate in this study.

## Author Contributions

WX: Conceptualization, methodology, investigation, visualization and writing-original draft. HC: Resources and Investigation. YF: Resources and Investigation. NS: Resources, data curation, and investigation. ZH: Formal analysis. QF: Resources and Investigation. XJ: Resources and investigation. GH and MX: Methodology and validation. YL and ZW: Methodology and resources. AT and XY: Supervision, project administration and funding acquisition. All authors contributed to the article and approved the submitted version.

## Funding

This study was supported by the National Natural Science Foundation of China (grants 32060132, 81760189, and 81760188), Guangxi Clinic Medicine Research Center of Nasopharyngeal Carcinoma (grant GuikeAD20297078), Guangxi Natural Science Foundation (No.2020GXNSFAA297235), and Innovation Project of Guangxi Graduate Education (grant YCBZ2020050).

## Conflict of Interest

The authors declare that the research was conducted in the absence of any commercial or financial relationships that could be construed as a potential conflict of interest.

## Publisher’s Note

All claims expressed in this article are solely those of the authors and do not necessarily represent those of their affiliated organizations, or those of the publisher, the editors and the reviewers. Any product that may be evaluated in this article, or claim that may be made by its manufacturer, is not guaranteed or endorsed by the publisher.
