# Supplementary figures and images for: Tree Shrew Is a Suitable Animal Model for the Study of Epstein Barr Virus
Source: Front Immunol. 2022 Jan 17;12:789604. doi: 10.3389/fimmu.2021.789604 (PMC8801525; doi:10.3389/fimmu.2021.789604)

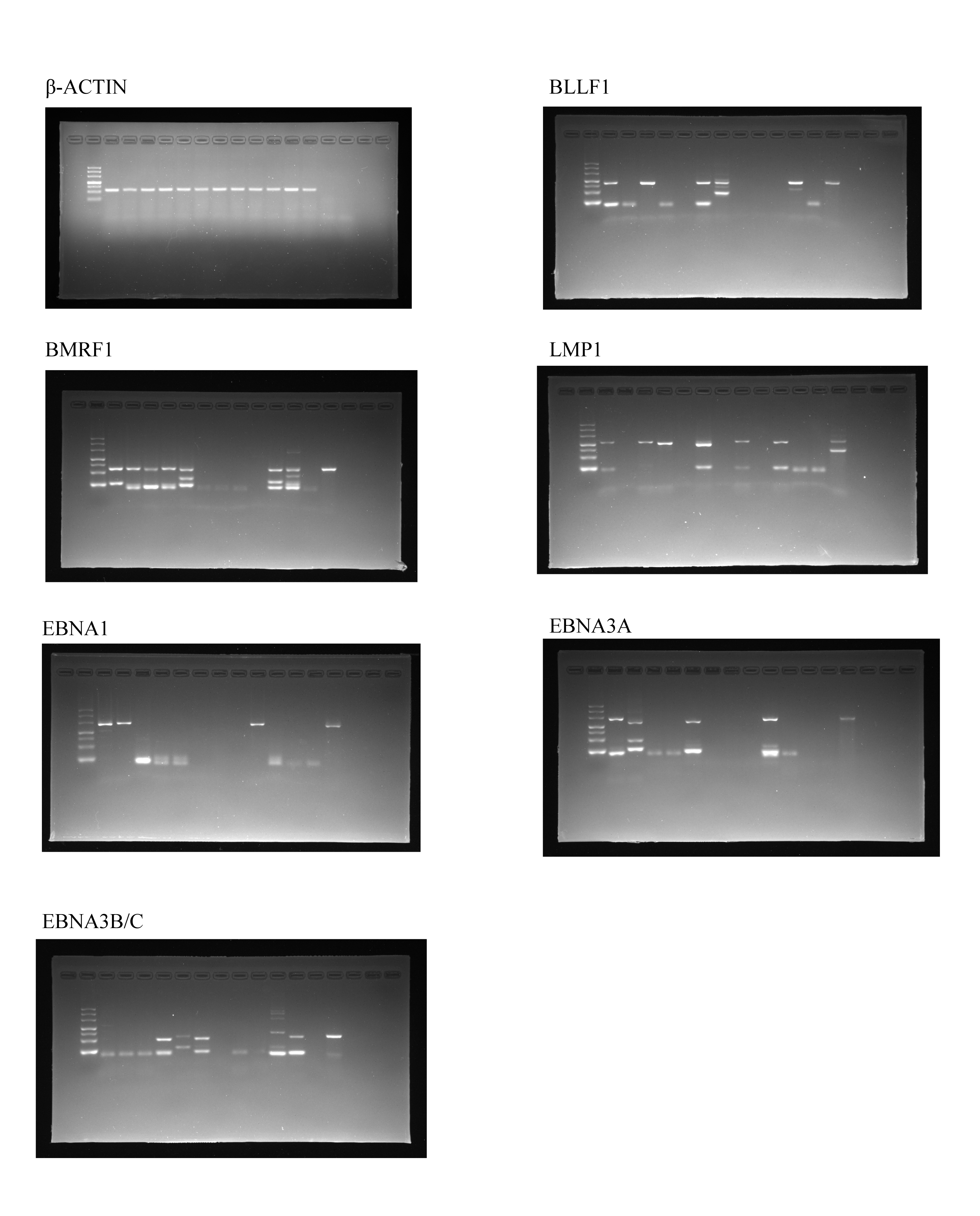

Supplement: Supplementary file 2 [file Image_1.tif]

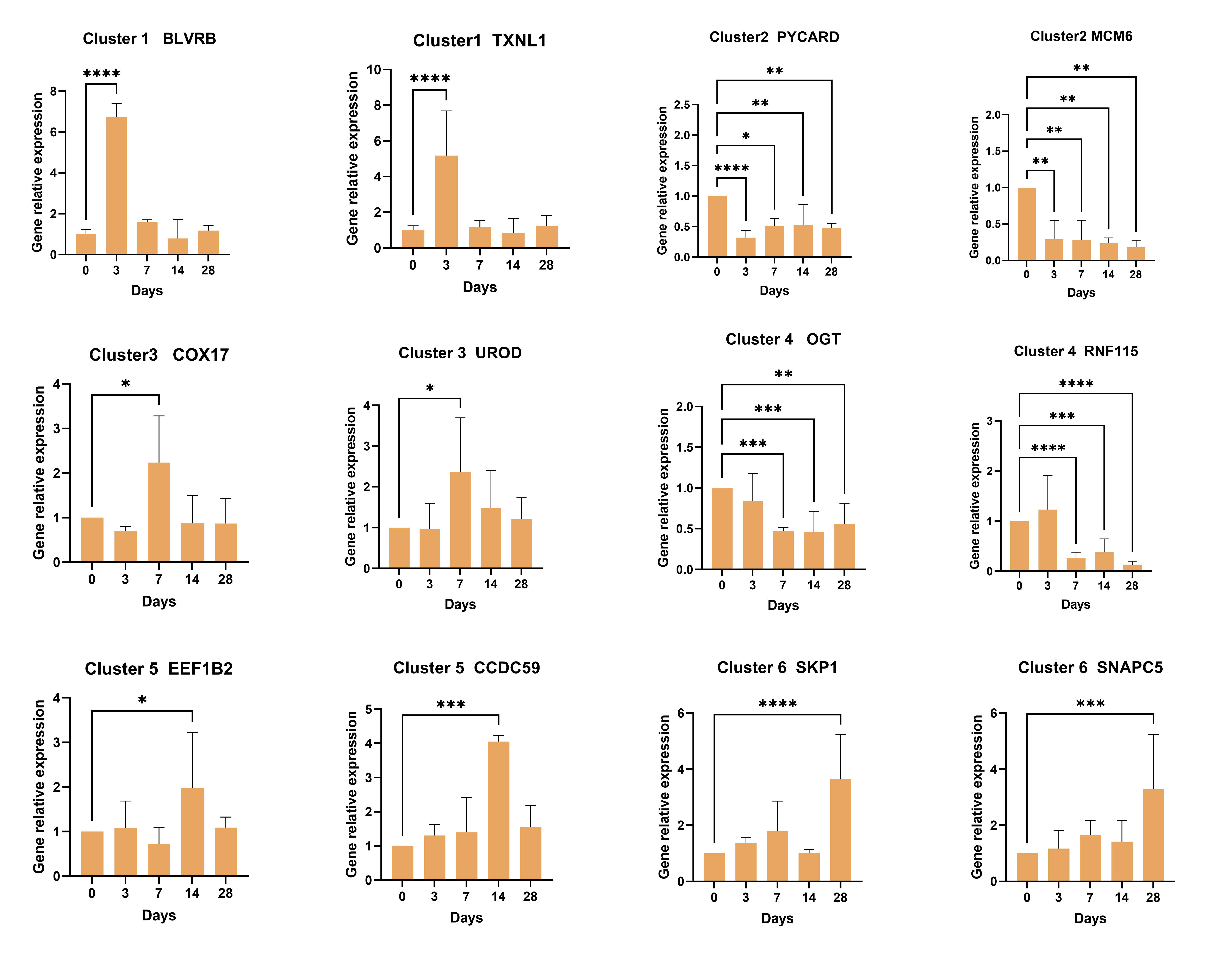

Supplement: Supplementary file 3 [file Image_2.tif]

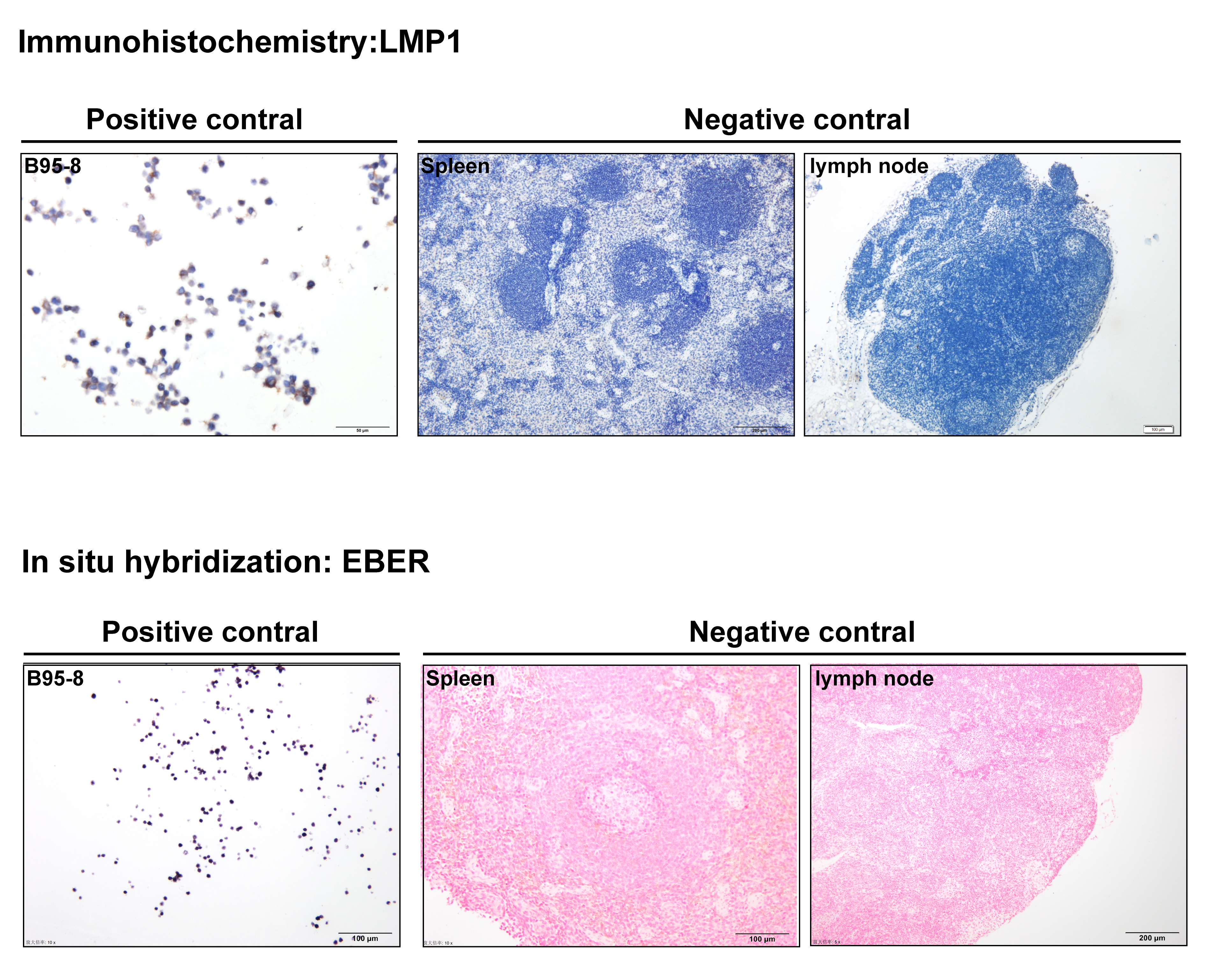

Supplement: Supplementary file 4 [file Image_3.tif]
